# Quadricuspid aortic valve repair: Tricuspidization with a double-ring annuloplasty

**DOI:** 10.1016/j.xjtc.2026.102314

**Published:** 2026-03-19

**Authors:** Margaux Bernardini, Claudia Côté, Pavel Zacek, Greg Hirsch, Pascal Leprince, Emmanuel Lansac, Pichoy Danial

**Affiliations:** a Department of Cardiovascular and Thoracic Surgery, Sorbonne University, Institute of Cardiology, Pitié-Salpêtrière Hospital, Assistance Publique-Hôpitaux de Paris (AP-HP), Paris, France; b Division of Cardiac Surgery, Department of Surgery, Halifax Infirmary, Halifax, Nova Scotia, Canada; c Department of Cardiac Surgery, Charles University, Faculty of Medicine in Hradec Kralove and University Hospital in Hradec, Kralove, Czechia

**Figure:**
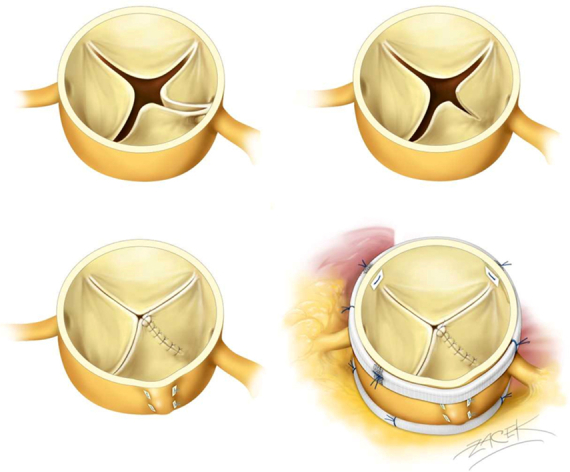
Tricuspidization with sinus plication and double ring annuloplasty.

**Central Message**

Tricuspidization provides a standardized approach of repair addressing the valve lesion without using a pericardial patch.

A 38-year-old patient was admitted for dyspnea and New York Heart Association functional class II. Echocardiography showed severe aortic insufficiency with dilation of the left ventricle (left ventricular end-diastolic diameter = 68 mm; left ventricular end-systolic diameter = 45 mm). Left ventricular ejection fraction was preserved at 67%. The valve was quadricuspid with 2 major cusps: noncoronary cusp and right coronary cusp, and one left coronary cusp divided in 2 hemi-cusps by a pseudocommissural raphe (Figure 1, *A*). There was a severe central aortic insufficiency as a result of the central diastasis related to retraction of the pseudocommissural raphe separating the 2 left hemi-cusps.

**Figure 1:**
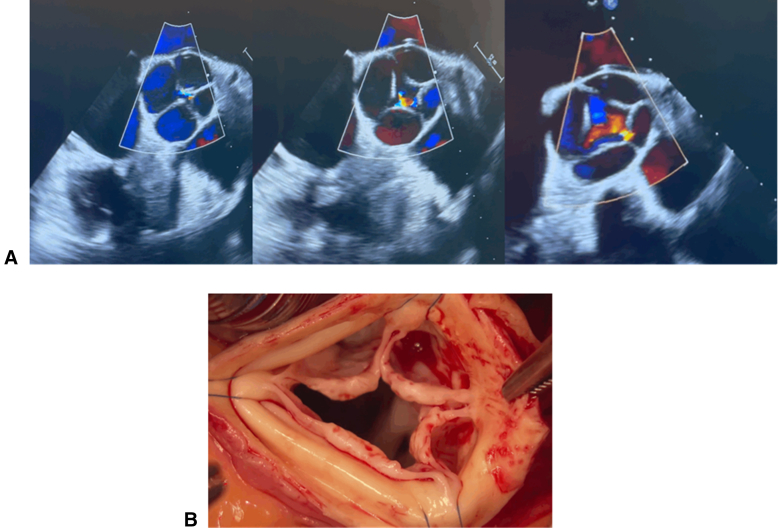
Preoperative transthoracic echocardiography (A) and surgical (B) view.

On the computed tomography scan, the maximal diameter of aortic annulus was 28 mm, the diameter of the sinotubular junction (STJ) was 38 mm, and the diameter of the tubular aorta was 42 mm. Valve-sparing tricuspidization of this quadricuspid aortic valve with double external ring annuloplasty was performed.

## Methods

This study was approved by the AVIATOR registry review boards (NCT00478803, May 25, 2007), which allows patients undergoing valve-sparing surgery to have deidentified intraoperative and postoperative follow-up details recorded and published for research purposes. Individual patient consent was waived. The procedure was performed via median sternotomy on cardiopulmonary bypass using standard techniques. The aorta was opened and the valve analyzed (Video 1). We confirm the quadricuspid nature of the aortic valve with the left coronary cusp divided in 2 hemi-cusps by a pseudo commissural raphe (Figure 1, *B*). A 30-mm Hegar dilator was passed easily, making the native annulus >30 mm.

Deep external dissection of the aortic root down to the subvalvular plane was performed, passing below the coronary arteries without detaching them. Five pledgeted 2-0 polyester braided mattress stitches were placed circumferentially at the subvalvular level for the ring annuloplasty, 3 below the nadir of each cusp and 2 at the base of each interleaflet triangle. An additional stitch was placed at the right and noncoronary commissure externally to avoid a potential injury to the bundle of His.

**Figure 2:**
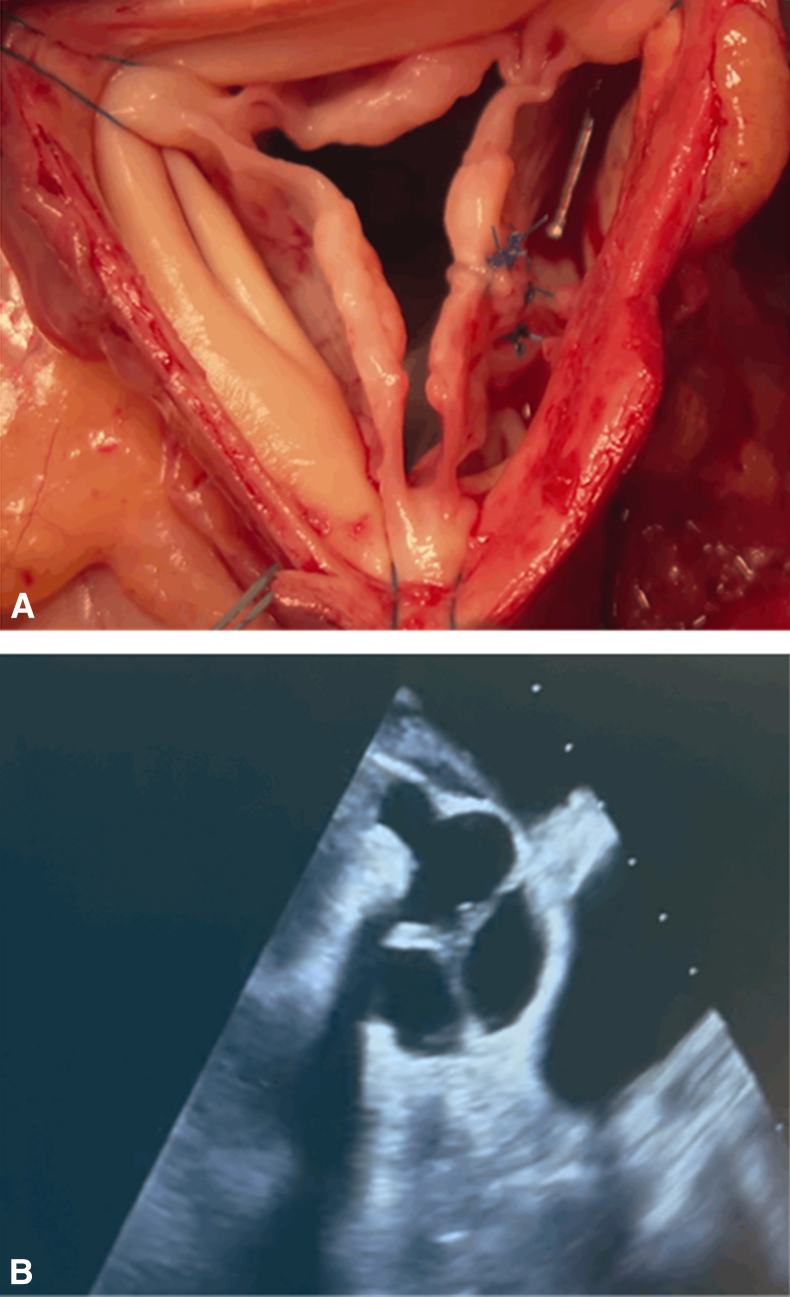
Postoperative surgical (A) and transthoracic echocardiography (B) view.

Alignment of the free-margin lengths was then performed to ensure a symmetrical valve. A 5-0 polypropylene suture was passed through each nodule of Arantius. Excess length of free the edge was then determined. The right coronary and noncoronary cusps had equal length. The pseudo-commissure was taken down to the annulus and the divided leaflet was reapproximated with interrupted 5-0 polypropylene sutures, to the same length as the reference cusps. The free margin length was then rechecked and an additional plication stich is taken to ensure 3 equal free margin lengths (Figure 2, *A*). A plication of the left sinus was added to improve the symmetrical design of the repair using two 2-0 polyester braided mattress sutures.

Circumferential reduction of the STJ was performed by placing five 2-0 polyester braided pledgeted mattress sutures, one above each commissure and one above each coronary ostium. The STJ stitches were then passed through a 29-mm expansile EXTRA-AORTIC ring (CORONEO Inc), with the commissures placed at 120 degrees in a tricuspid configuration. Next, the effective height of each cusp was checked using a cusp caliper. The external annular ring was fashioned using an open piece of 30-mm Dacron tube graft placed under the coronary arteries, and the subvalvular stitches were passed through and tied. Finally, the aortotomy was closed using a polypropylene 4-0 running suture.

## Results

Final echocardiography showed satisfactory repair outcomes with no residual regurgitation and a mean aortic valve gradient of 2 mm Hg (Figure 2, *B*). At 1 month postoperatively, the patient had with similar findings on echocardiogram. The patient was asymptomatic, in sinus rhythm.

## Discussion

The major advantage of the technique presented in this article is the absence of the use of a pericardial patch. Several tricuspidization techniques described required the use of a pericardial patch, which is a known risk factor for reintervention.^1^, ^2^, ^3^ We advocate for systematic STJ stabilization to maintain STJ to annulus ratio and valve durability over time.^4^ This case demonstrates options for repair provided adequate leaflet tissue for a durable repair.

## Conclusions

Tricuspidization of quadricuspid aortic valve with double-ring annuloplasty provides a standardized approach of repair addressing valve lesion and annulus dilation without using a pericardial patch.

## Conflict of Interest Statement

Dr Lansac has consultant agreements with CORONEO, Inc. All other authors reported no conflicts of interest.

The *Journal* policy requires editors and reviewers to disclose conflicts of interest and to decline handling or reviewing manuscripts for which they may have a conflict of interest. The editors and reviewers of this article have no conflicts of interest.

## Supplementary Data

Video 1 Operative technique of tricuspidization of quadricuspid aortic valve. Video available at: https://www.jtcvs.org/article/S2666-2507(26)00121-5/fulltext.
