# Supplementary figures and images for: Quadricuspid aortic valve repair: Tricuspidization with a double-ring annuloplasty
Source: JTCVS Tech. 2026 Mar 19;37:102314. doi: 10.1016/j.xjtc.2026.102314 (PMC13261192; doi:10.1016/j.xjtc.2026.102314)

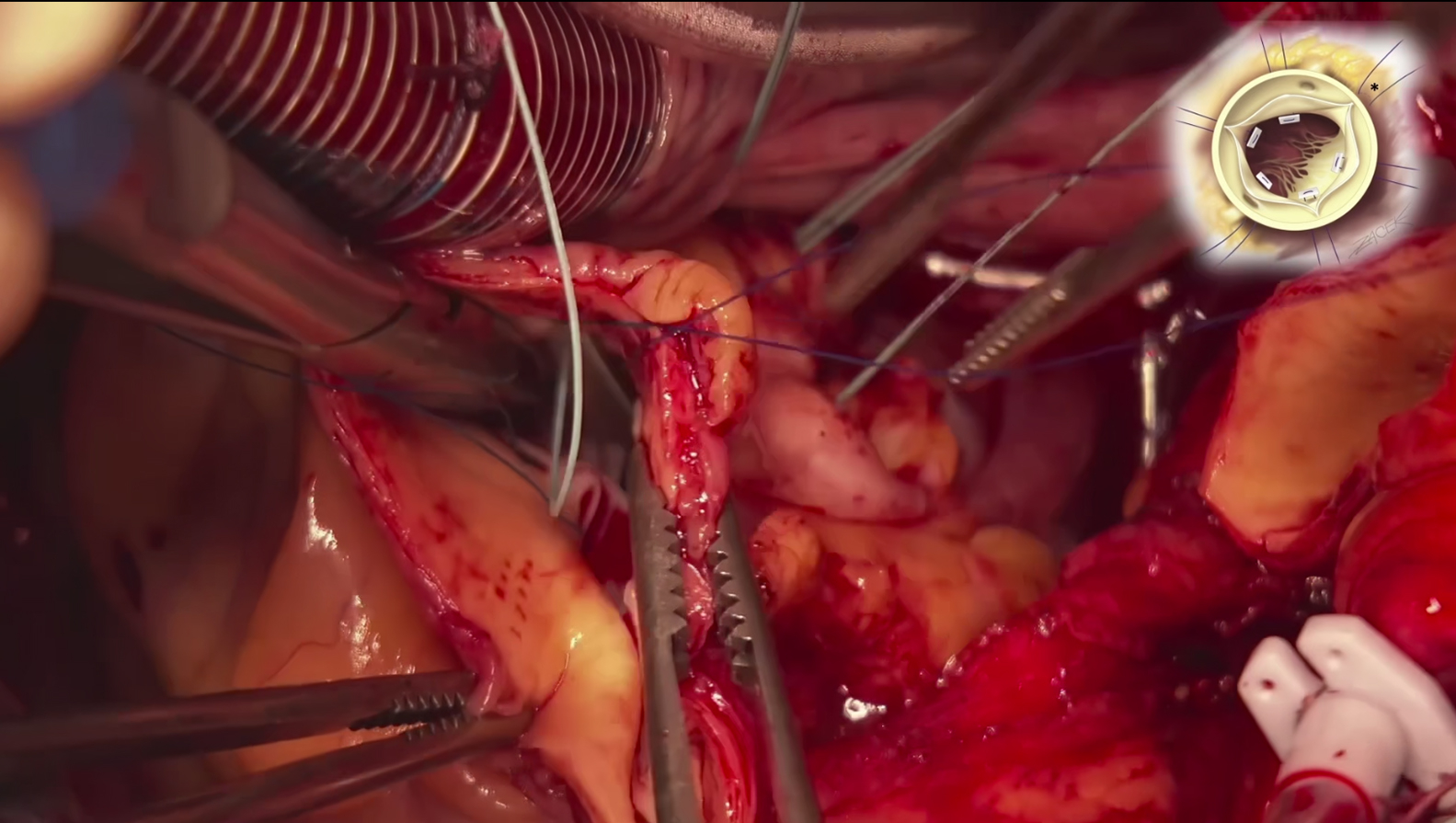

Supplement: Video 1 — Operative technique of tricuspidization of quadricuspid aortic valve. Video available at: https://www.jtcvs.org/article/S2666-2507(26)00121-5/fulltext. [file fx2.jpg]
